# Combinatorial Action of miRNAs Regulates Transcriptional and Post-Transcriptional Gene Silencing following *in* *vivo* PNS Injury

**DOI:** 10.1371/journal.pone.0039674

**Published:** 2012-07-06

**Authors:** Tadepalli Adilakshmi, Ida Sudol, Nikos Tapinos

**Affiliations:** Molecular Neuroscience Laboratory, Weis Center for Research, Geisinger Clinic, Danville, Pennsylvania, United States of America; George Mason University, United States of America

## Abstract

Injury response in the peripheral nervous system (PNS) is characterized by rapid alterations in the genetic program of Schwann cells. However, the epigenetic mechanisms modulating these changes remain elusive. Here we show that sciatic nerve injury in mice induces a cohort of 22 miRNAs, which coordinate Schwann cell differentiation and dedifferentiation through a combinatorial modulation of their positive and negative gene regulators. These miRNAs and their targeted mRNAs form functional complexes with the Argonaute-2 protein to mediate post-transcriptional gene silencing. MiR-138 and miR-709 show the highest affinity amongst the cohort, for binding and regulation of Egr2, Sox-2 and c-Jun expression following injury. Moreover, miR-709 participates in the formation of epigenetic silencing complexes with H3K27me3 and Argonaute-1 to induce transcriptional gene silencing of the Egr2 promoter. Collectively, we identified a discrete cohort of miRNAs as the central epigenetic regulators of the transition between differentiation and dedifferentiation during the acute phase of PNS injury.

## Introduction

Specific patterns of gene expression ensure the proper control of cognitive, behavioral, motor and sensory functions of specialized cells like nerve and glial cells. Yet, insults like nerve injury, neurodegenerative diseases, infection, and demyelinating diseases can affect nerve cells, mobilizing a “reprogramming process” that may be able to reset previously established cellular settings and dedifferentiate these cells to a more primitive state. How could one relatively fixed genetic blueprint permit this flexibility? One way that such additional variability can be established is through epigenetic mechanisms [1]. It has been shown that Schwann cells (the glial cells of the PNS) retain a remarkable plasticity that allows them to dedifferentiate after peripheral nerve injury, shed off the specialized myelin sheath, participate in myelin debris clearance and eventually drive nerve regeneration and re-myelination [2], [3]. However, the underlying epigenetic mechanisms responsible for this flexibility in regulating gene expression patterns after nerve injury of the PNS remain elusive.

Mammalian cells produce a class of non-coding RNAs, microRNAs (miRNAs) in a cell-type-specific manner and utilize them for acute and rapid regulation of gene expression by various mechanisms such as transcriptional gene silencing [4], [5], post-transcriptional gene silencing [6], [7] and epigenetic modifications [8], [9]. An RNase III enzyme, Dicer, processes miRNAs into ∼ 22 nucleotide long mature RNA duplexes [10] that are then loaded into the RNA-induced silencing complex (RISC), which contains Argonaute (Ago) proteins [11], [12]. MiRNAs function as probes to target multiple mRNAs leading to translational repression or, sometimes, to mRNA degradation [13].

Here, we report that a specific cohort of miRNAs controls directly or indirectly the expression of positive and negative regulators of myelination and dedifferentiation such as Egr2, c-Jun, Sox2, Nanog, ID2, p75, QKI-6 through acute post-transcriptional gene silencing after PNS injury *in vivo*. MiR-138 and miR-709 show the highest affinity for binding and regulation of Egr2, c-Jun and Sox-2 expression, which are the main gene regulators of the transition between differentiation and dedifferentiation following PNS injury [14]. We also demonstrate that miR-709 is involved in regulating transcriptional gene silencing of Egr2, a master regulator of the dedifferentiation/myelination switch [15]. The transcriptional silencing is accomplished through direct interaction of miR-709 with the myelin specific element (MSE) of the Egr-2 promoter, which affects nascent transcription of Egr2 and through the formation of epigenetic silencing complexes comprised of the repressive histone mark H3K27me3, Ago-1 and miR-709 on Egr2 promoter. Collectively, we implicate miRNAs as central epigenetic regulators of the transition between differentiation and dedifferentiation of Schwann cells during the acute phase of injury response in the PNS.

## Results

### Expression of miRNAs and Schwann Cell Dedifferentiation

**Figure 1:**
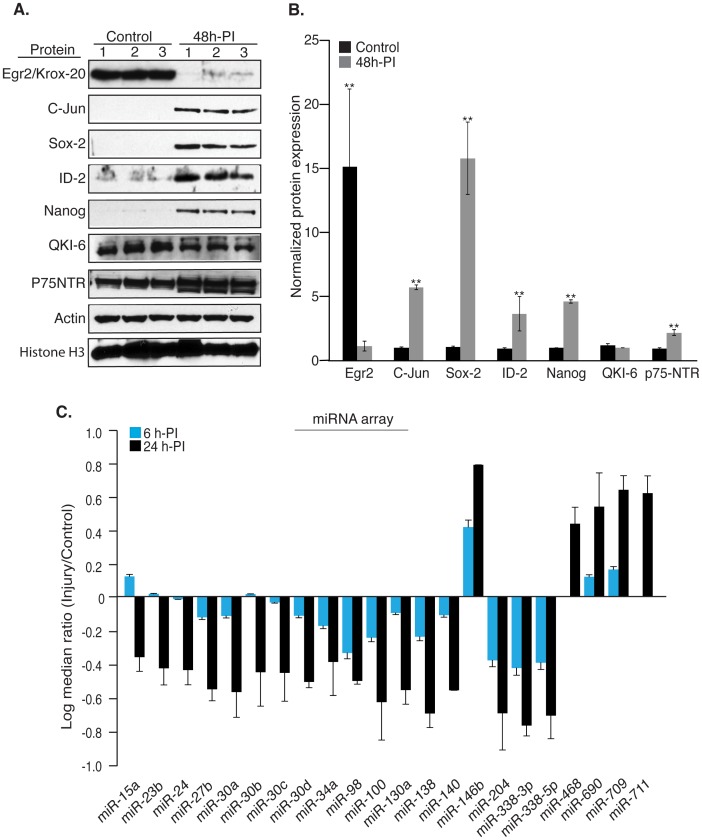
Protein expression changes and miRNA expression profile following *in vivo* sciatic nerve injury. Protein expression analysis reveals a robust reduction in pro-myelination and upregulation of anti-myelination factors. All experiments were repeated three times unless otherwise stated. (A). Uninjured and distal segments of axotomized sciatic nerves at 48 hours post injury (PI) (n = 4) were lysed in SDS buffer and western blots were performed for Egr2 (63 kDa), C-Jun (43 kDa), Sox-2 (35 kDa), ID-2 (15 kDa), Nanog (34–40 kDa), QKI-6 (38 kDa), P75^NTR^ (75 kDa), beta-actin (42 kDa) and Histone H3 (17 KDa). (B). Relative protein levels normalized to beta-actin were plotted to demonstrate the differential expression of the pro-myelination and anti-myelination factors in injury. Statistical significance was calculated with a Student’s t-test (**: p<0.001). (C). Microarray for miRNAs (version 9.2) was performed using total RNA from 15 control and 15 axotomized mouse sciatic nerves at 6 hours and 24 hours post-injury (PI), by Exiqon (Vedbaek, Denmark). The array was repeated twice and Log2 median ratios of the miRNAs expressed in 24h-injured nerve compared to the control were plotted and the standard error was calculated.

Axotomy changes Schwann cell morphology from a mature, myelinating phenotype to an immature, regeneration supporting phenotype, a process exactly opposite to that observed during development [16]. Schwann cells contain both negative and positive transcriptional regulators of myelination that functionally complement each other [14]. The response of Schwann cells to injury is therefore determined by a delicate balance between these two opposing transcriptional programs. To demonstrate this in the context of *in vivo* sciatic nerve injury, we examined the expression of certain promyelinating and anti-myelinating factors such as Egr2/Krox-20, QKI-6, Id2, Sox-2, c-Jun and P75^NTR^. We also probed for the expression of the stem cell marker Nanog since cells exiting differentiation should express it [17]. Interestingly, there is a sharp change in the balance between these factors within 48 hours after injury (Fig. 1A & B). While the expression of the pro-myelinating factor Egr2 is repressed, antimyelination/dedifferentiation factors such as Id2, Sox-2 and c-Jun were upregulated. Accordingly, Nanog was also significantly upregulated upon injury suggesting activation of a dedifferentiation/reprogramming phenotype. Following nerve injury QKI is post-translationally modified as observed by the shift in the protein band (Fig. 1A), while p75^NTR^ is up-regulated as indicated by the unmodified nascent protein (∼50 KDa, lower band in P75NTR blot, Fig. 1A). Finally, we examined the expression of beta-actin and histone H3 to normalize our results. Beta-actin is expressed in both axons and non-neuronal cells while histone H3 is a non-axonal protein that shows relatively little change in the nerve at 48 h after injury.

The rapid change in the expression of various proteins observed 48 hours after peripheral nerve injury (Fig. 1A) suggests a central epigenetic mechanism responsible for a synchronized regulation of multiple targets. We hypothesized that miRNAs modulate the observed protein output in injury. As an important component of gene regulation in higher eukaryotes, miRNAs bind the effector protein Argonaute (Ago) to form the mature silencing complexes that elicit silencing of several mRNA targets [18]. To understand the interplay among miRNAs in fine-tuning the dynamic changes that occur during the acute phase of PNS injury, we assayed their fundamental characteristics including their expression pattern, gene targets and phenotypic synergism. In mice lacking the ribonuclease Dicer in Schwann cells, PNS myelin ablation precedes axonal degeneration [19], [20]. Similarly, following nerve injury the Schwann cell response precedes axonal degeneration [21]. Consistent with this time frame and based on the protein expression changes, we examined the miRNA expression profile at 6 hours and 24 hours after sciatic nerve injury and compared with an uninjured control (Exiqon Inc.). This analysis identified 22 miRNAs that are differentially regulated in injury as early as 6 hours and persisted until 24 hours post injury (Fig. 1C). We confirmed the miRNA expression profile with real-time RT-PCR at 24 hours post-injury for all the miRNAs that had validated probes available (Fig. S1). Expression of a few miRNAs that differed maximally in the miRNA array, namely 138, 690 and 711 was further confirmed using Northern blot analysis (Fig. S2).

The expression pattern of miRNAs in injury is in agreement with previous studies that reported the importance of miRNAs for Schwann cell differentiation and myelination [20], [22], [23]. For instance, miRNAs that were significantly upregulated in sciatic nerves during myelination and were considerably repressed by Schwann cell specific deletion of Dicer such as 23b, 24, 27b, 34a, 100, 138, 140, 338-3p [20], [22], [23] were repressed upon injury with the exception of miR-146b (Fig. 1C). Repression of this set of miRNAs indicates that the Schwann cells significantly contributed to the miRNA cohort in injury. Conceivably, the repression of those miRNAs that normally suppress immature Schwann cell phenotype should lead to Schwann cell dedifferentiation in injury. However, to ensue dedifferentiation, ongoing myelination process has to be terminated and we hypothesize that the cohort of miRNAs (146b, 468, 690, 709 and 711) that are upregulated in injury might function in reverting the myelination/differentiation phenotype.

### Prediction and Evaluation of miRNA Binding Sites in Targets

To understand the complexity of the miRNA-mRNA interplay and to identify if the protein expression pattern following sciatic nerve injury (Fig. 1A) can be attributed to miRNA targeting we employed several computational analysis tools. Since miRNAs co-target many genes both within and between pathways [24], delineating their cellular function depends on identifying *bona fide* targets for the cohort of miRNAs. Attempts to identify the interaction of miRNAs with their targets are confined to an underlying assumption that 3′-untranslated regions [7] of mRNAs are the principle recipients of miRNA activity. However, recent studies[25]–[27] have reported that miRNA targeting can occur in coding sequences as well. Another extensively discussed issue in the miRNA literature is the question of the typical number of functional targets per miRNA and the related question of what fraction of seed matches correspond to functional target sites. Moreover, free energy of miR:mRNA duplex, binding site accessibility, and target structure influence the efficiency of a miRNA:mRNA functional interaction, in addition to the conserved seed-region [28].

**Figure 2:**
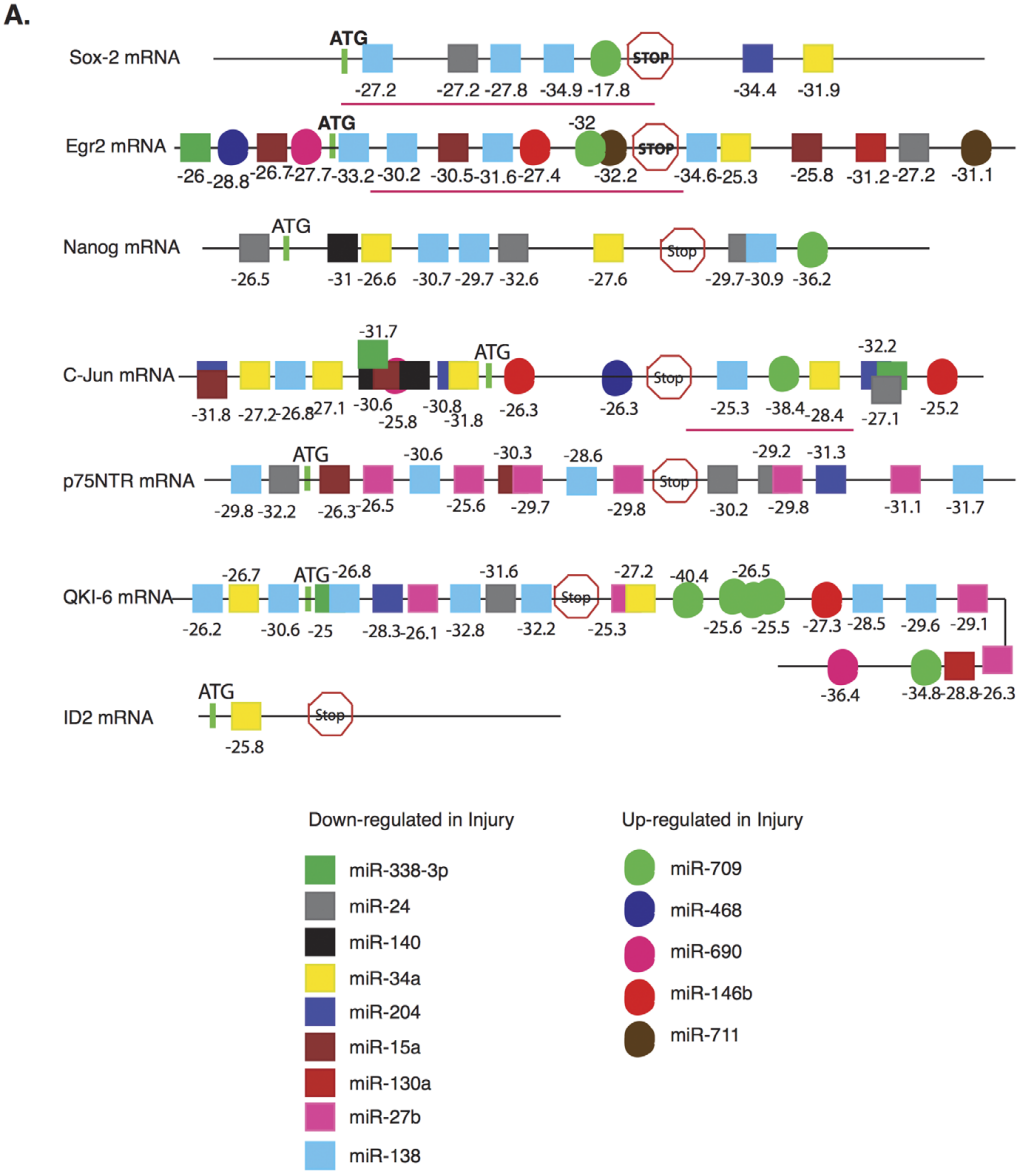
miRNA-mRNA target prediction and evaluation. Putative miRNA binding sites detected by RNA 22 are represented on individual mRNAs (not to scale) as squares (repressed in injury) or circles (up regulated in injury) with the maximum folding energy shown below. Underlined areas indicate regions used in Luciferase assays.

Considering all these factors we employed two independent methods of computational prediction analysis: RNA 22 [29] and sfold STarMir [28] to underscore the contribution of individual and clusters of miRNAs to the injury response process. RNA22 enables identification of miRNA(s) interactions with gene sequences beyond the confines of 3′UTR. RNA 22 analysis of particular genes with a central role in injury response like Egr2/Krox-20, Id2, Sox-2, Nanog, c-Jun as well as p75^NTR^ and QKI (Fig. 1A), predicted an abundance of targets for our miRNA cohort in the coding sequences and the UTR regions (Fig. 2).

Sequence context influences miRNA efficacy by mediating the binding of hypothetical cofactor proteins or by affecting the secondary structure of a target site and hence its accessibility to binding by the miRNA [30]. STarMir analyzes the accessibility of each of these predicted putative miRNA binding sites in individual messages in the context of its secondary structure and if the miRNA:mRNA complexes are functional (total energy of the hybrid ≤-10 kcal mol^-1^ means an efficient interaction). STarMir analysis identified multiple sites for each miRNA of our cohort (miRNA 138 and 709 on Egr2, Sox-2 and c-Jun are shown: Table 1) with variable functional efficacies as evidenced by the total energy of the miRNA-mRNA hybrid (results for all the members of our miRNA cohort in Table S1). Although all the genes analyzed exhibit functionally efficient miRNA binding sites some of these sites are mutually exclusive indicating a competitive binding mechanism among miRNAs during the acute phase of PNS injury response. Alternatively, unknown factors binding to neighboring RNA elements help achieve interaction specificity differently for each individual mRNA in a manner independent of site types. Therefore, it is possible that multiple components in various pathways are modulated by clusters of our cohort of miRNAs resulting in a modulation of target genes with a crucial role in injury response. To fully realize this potential we need to understand how miRNAs function singly and in concert with each other.

**Table 1:** STarMir analysis of accessibility of miRNA binding sites in targets (ΣΔG total, Kcal/mol).

Gene	miR-138	miR-709
Egr2	−14.79	−19.8
	−14.26	−16.5
	−13.89	−13.8
	−12.25	−13.48
	−11.8	−12.84
		−12.82
		
Sox-2	−11.2	−9.95
	−5.02	
	−4.58	
	−3.54	
		
C-Jun	−9.98	−14.35
	−9.42	−13.94
		−11.12
		−10.06

A value less than -10 Kcal/mol means efficient interaction.

### miR-138 and miR-709 Bind and Regulate the Expression of Sox-2, c-Jun and Egr2

**Figure 3:**
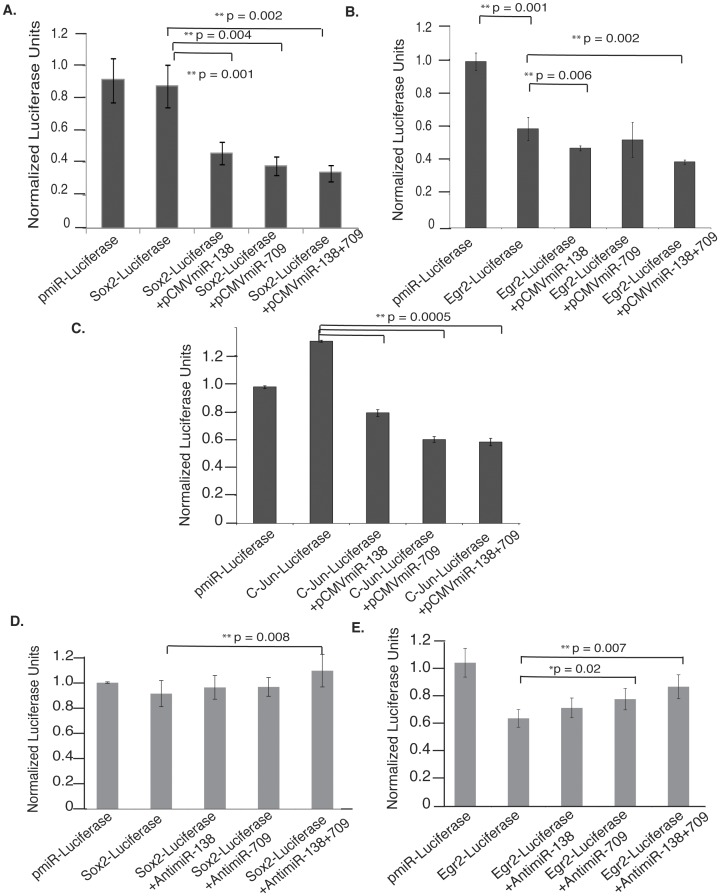
miR-138 and miR-709 bind and regulate Sox-2, c-Jun and Egr2. Sox-2, c-Jun and Egr2 transcripts containing multiple target sites for both miRNAs 138 and 709 were cloned into the 3′-UTR of luciferase gene in the pmiR-report vector. Cos-7 cells were co-transfected with miR-target vector and pmir-Report β-Gal vector as a control for transfection efficiency. Cell lysates were assayed 24 h post transfection for luciferase and β-Gal expression and β-Gal is used to normalize for differences in transfection efficiency. Sox-2, which lacks highly accessible sites for miR-138 and miR-709 show comparable expression to control (A & D, first two bars), while c-Jun (C, first two bars) and Egr2 (B & E, first two bars) that have multiple highly accessible sites for both miR-138 and 709 (Table 1 & Table S2) are highly de-repressed (c-Jun) or highly repressed (Egr2) by endogenous miRNAs. When miRNAs were overexpressed in molar excess through transfection, then Sox-2, c-Jun and Egr2 constructs were significantly repressed (A - E, last three bars). Luciferase assays with the available mouse anti-miRs for miR-138 and 709 again revealed significant derepression by individual anti-miRs for Sox-2 and Egr2 (D & E,). The combinatorial effect of both miRNAs is more significant for all three genes (A-E, last bar). ** denotes statistical significance (p<0.005) as calculated by Student’s t-test.

Sox-2, c-Jun and Egr2 mRNA transcripts with natural miRNA binding sites in the context of their respective secondary structure (cloned regions are underlined in Fig. 2) were analyzed using luciferase assays in order to verify the binding efficiency and regulation by miRNAs. We selected Sox-2, c-Jun and Egr2 for two reasons: First their role as central mediators of dedifferentiation and myelination/demyelination is established by independent studies [14]. Second, our computational analysis of miRNA predicted targets show that these three genes have multiple sites for regulation by our miRNA cohort (Fig. 2 and Table S1). We selected miR-138 and miR-709 for exogenous expression as Sox-2, c-Jun and Egr2 had multiple sites for these two miRNAs (Fig. 2, Table 1). STarMir analysis of the gene constructs showed few differences in the accessibility of sites when compared to the complete mRNA (Table S2). Moreover, constructs were selected with multiple putative seed regions, which were positioned in the same relative locations in each vector ensuring no proximity to the ends of the 3′-UTR. In addition, we wanted to use two miRNAs with opposing function in PNS injury (miR-138 is down-regulated while miR-709 is up-regulated) to emphasize that the final outcome of gene expression is molded by a synchronized action of opposing signals. The end-result (repression or de-repression of expression) depends on the availability of the target sites, secondary structure and the role of co-factors. In this context, although Sox-2, c-Jun and Egr2 show binding sites for both miR-138 and 709 the repression of the transcripts by endogenous miRNAs vary as compared to luciferase control (Fig. 3A-E, first two bars). This is consistent with the accessibility of the sites in individual RNA (STarMir, Table 1 & S2). For instance, Egr2 that has multiple highly accessible sites for both miR-138 and 709 (Table 1) is repressed by endogenous miRNAs (Fig. 3B & 3E first two bars), while Sox-2 and c-Jun with less accessible sites were comparable to the control or even up-regulated (Fig. 3A – 3D, first two bars). Albeit, when miRNAs were overexpressed in molar excess through transfection, then Sox-2 and c-Jun constructs were also repressed (Fig. 3A – 3E). Luciferase assays with the available mouse anti-miRs for miR-138 and 709 again revealed significant derepression by individual anti-miRs for Sox-2, c-Jun and Egr2 (Fig. 3A – 3E last three bars) verifying the specificity of the miRNA action. The combinatorial effect of miR-138 and miR-709 was validated as shown in Fig. 3A-E (last bar). Cumulative inhibition by both miRNAs resulted in a more significant decrease in the luciferase activity for both genes (Fig. 3A-C). Similar results were obtained with anti-miRs inhibiting both miRNAs (Fig. 3D & E). Finally, to show that the addition of the CMV-miR vector has no effect on luciferase expression in the empty sensor vector we analyzed the effect of the pCMV-miRNA expressing vectors, as well as miR-709 and miR-138 on the expression of luciferase from pMIR-report vector. As shown in Fig. S3, when pMIR-luciferase vector was co-transfected with either pCMV-empty vector, or pCMV-miR-709 or pCMV-miR-138 and b-gal transfection control vector the expression of luciferase was not significantly affected. In conclusion, our Luciferase expression data demonstrate that miR-138 and miR-709 can efficiently bind and regulate the expression of Sox-2, c-Jun and Egr2 in the context of an in vitro experiment.

### miRNAs Form Functional Complexes with Their Targeted mRNAs in Association with Ago-2 *in vivo*

**Figure 4:**
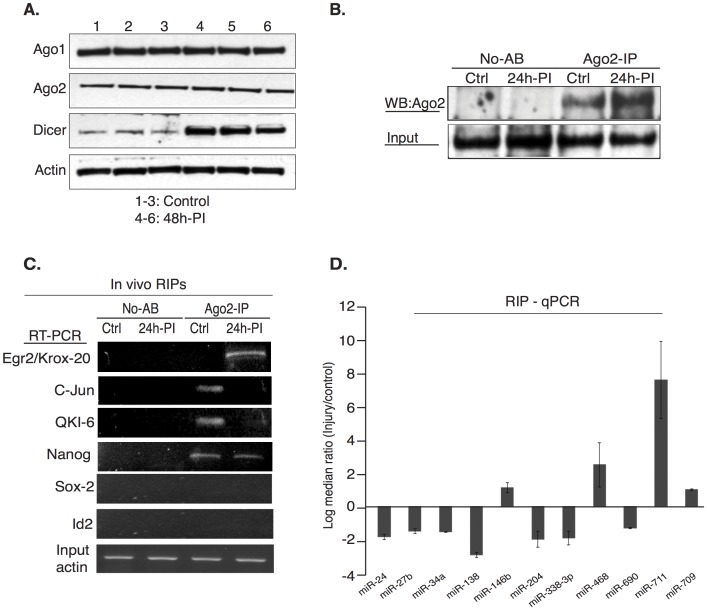
miRNAs induce post-transcriptional gene silencing through association with Ago-2 in functional complexes. (A). Protein expression of Ago-1, Ago-2 and Dicer in sciatic nerves before and after nerve injury to confirm the expression of the miRNA processing machinery proteins. Actin was used as a loading control. (B) Cytoplasmic lysates isolated from control sciatic nerves and injured distal segments (10 nerves each) were immunoprecipitated with Ago-2 antibody (Cell Signaling, USA) or IgG control. A portion (1/3^rd^) of the sample was used for each of the analysis and the experiment was repeated twice. Input, No-Antibody (No AB) and Ago-2 immunoprecipitated protein was analyzed by western blotting with Ago-2 antibody, which shows enrichment of Ago-2 in the 24 hour post-injury samples. (C). For RNA-IPs (RIPs), mRNAs that were co-immunoprecipitated with Ago-2 *in vivo,* were reverse transcribed using oligo-dT primer and genes of interest were PCR amplified using gene-specific primers (Krox-20  =  1274 bp, C-Jun  =  689 bp, Nanog  =  753 bp, QKI-6  =  1345 bp, Sox-2  =  958 bp and ID-2  =  588 bp). (D). microRNAs that were co-immunoprecipitated with Ago-2 in complex with their targeted mRNAs, were reverse-transcribed with microRNA specific RT-primers using Multiscribe RT kit. Ago-2 associated microRNAs were detected by real time qPCR with miRNA specific Taqman probe/primer sets. Data were normalized to input and an internal control. Fold difference (2^-ΔΔCT^) in the association of individual microRNAs with Ago-2 protein between injured and control nerves was plotted as log 2 median ratio and error is expressed as standard deviation.

Our results show that the protein expression pattern during peripheral nerve injury response (Fig. 1A) is a result of the synchronous and concerted action of miRNAs with similar or opposing roles (e.g. miR-138 and miR-709). To reveal the mechanism for the robust effect on post-transcriptional gene silencing we examined the association of miRNAs and the transcripts of interest with Argonaute-2 (Ago2) protein *in vivo* after sciatic nerve injury. We also examined the expression of the components of miRNA machinery such as Dicer, Ago1 and Ago2 upon injury. Dicer is reported as a stress response component and that differences in Dicer protein levels may be reflected in the constitutive levels of cellular miRNAs [31]. Interestingly, Dicer is induced in injury while Ago1 and Ago2 remain unchanged suggesting that processing of miRNA increases while different set of miRNAs are guided by Argonaute proteins in injury (Fig. 4A). miRNAs guide members of the Ago protein family to partially complementary sequences within the target mRNAs [32], [33] via Watson-Crick base-pairing between the 5′-end of the miRNA and a target message to elicit translational repression or mRNA decay [18], [34]. Using specific monoclonal antibody against Ago2, we immunoprecipitated Ago2-mRNA-miRNA silencing complexes from sciatic nerves. Immunoprecipitated Ago2 protein was probed with Ago2 antibody to show the specificity of the antibody and co-immunoprecipitations with rabbit IgG were used as controls (Fig. 4B). Transcripts were amplified with gene-specific primers from Ago2 and IgG bound RNA using RT-PCR (Fig. 4C). Egr-2 and c-Jun transcripts were associated with Ago2 in a reciprocal manner consistent with their antagonistic roles in Schwann cells. On the other hand Sox-2 and Id2 were not detected with Ago2 in either condition. This could be explained by the lack of Sox-2 and Id2 transcripts in control myelinating sciatic nerves, while they are actively translated in injured nerves. In addition, Nanog is retained with Ago2-miRNA complex in injury while QKI-6, which also shares most binding sites is released from Ago2 (Table S1 & Fig. 4C). A detailed analysis revealed that binding sites for miRNAs 27b, 100, 140, 338-3p and 338-5p are much more efficient for QKI-6 when compared to Nanog (Table S1, in bold) and the repression of this set of miRNAs in injury signals the release of QKI transcript from Ago2 complex. On the other hand binding sites for miR-609 are stronger for Nanog, which may explain the recruitment of Nanog to Ago2 complexes in injury. Despite the association with Ago2 complex, Nanog is efficiently translated in injury, while QKI-6 is not (Fig. 1A). The regulation of QKI-6 occurs at post-translational level as observed by the shift in the protein band (Fig. 1A) consistent with reports that Src-PTK-mediated phosphorylation of QKI negatively regulates its RNA binding ability [35] resulting in the translational repression of Egr2, MBP and other targets.

Finally, to examine the formation of functional Ago2-mRNA-miRNA silencing complexes we amplified individual miRNAs that had available validated probes, from Ago2 co-immunoprecipitated RNA by real-time RT-PCR (Fig. 4D). The association of miR-709 with Ago2 was unperturbed suggesting that miR-709 may act on different subset of genes in normal and injured nerves to mediate post-transcriptional gene silencing or that miR-709 functions independent of Ago-2 association. Collectively, the real-time RT-PCR data show a decrease in the Ago2-association of miRNAs repressed in injury while the miRNAs upregulated in injury were increased in Ago2-complex (Fig. 4D) clearly indicating a correlation between expression and function.

### miR-709 Induces Transcriptional Gene Silencing of Egr2

**Figure 5:**
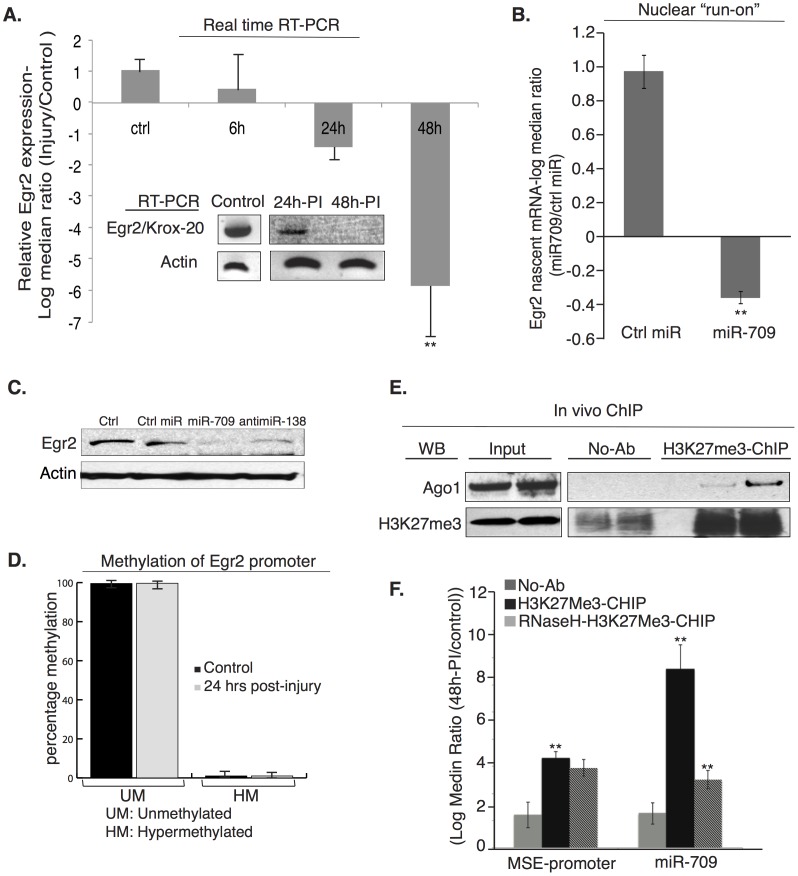
miRNAs mediate transcriptional gene silencing of Egr2. (A). Real-time qRT-PCR of Egr2 transcripts normalized to GAPDH control 6, 24 and 48 hours post-injury as compared to control uninjured nerves. Fold difference (2^-ΔΔCT^) between injured and control nerves is plotted and error is expressed as standard deviation. Inset: Egr2 mRNA expression at 24 hours and 48 hours after injury as compared to Egr2 mRNA expression in uninjured nerves. Egr2 mRNA expression is completely inhibited 48 hours post-injury. Beta-actin was used to show equal loading and amplification. (B). Nuclear “run-on” experiment for nascent Egr2 mRNA transcription in rat Schwann cells transfected with control or miR-709 duplexes as measured by quantitative RT-PCR and normalized to GAPDH mRNA transcription levels. The experiment was repeated three times, the data were normalized to GAPDH and fold difference (2^-ΔΔCT^), between injured and control nerves was plotted as a log-2 median ratio. Error is expressed as standard deviation, (**: p<0.005). (C). Egr2 protein expression in Schwann cells transfected with miR-709, antimiR-138 or non-targeting control miRNA and compared with non-transfected Schwann cells. (D). Methylation PCR analysis (SA Biosciences) of the CpG islands of the proximal Egr-2 promoter from control and injured sciatic nerves (24h-post injury). The relative percentages of hypermethylated (HM) and unmethylated (UM) fractions were calculated by comparing the amount in each digest with that of a mock (no enzyme added) digestion. (E). ChIP assays, using antibody to H3K27Me3 or no antibody (No Ab) controls were performed in sciatic nerves isolated from control and axotomized mice 48 hours post-injury. Western blot of input, no antibody control (No Ab) and H3K27Me3-ChIP was performed with antibodies against Ago-1 and H3K27Me3. This showed that Ago-1 is enriched in H3K27me3 silencing complexes following *in vivo* peripheral nerve injury. (F). Real-time quantitative PCR to assess the presence of the MSE region of the Egr2 promoter (left bars) and miR-709 (right bars) in the H3K27me3 immunoprecipitated chromatin. Lysates pre-treated with RNase H were immunoprecipitated to confirm RNA-DNA interaction. Values for the MSE and miR-709 were normalized to input DNA and miRNA respectively. The fold difference (2^-ΔΔCT^) in the association of MSE and miR-709 with H3K27Me3 complex between injured and control nerves, was plotted as a log-2 median ratio and the error is expressed as standard deviation. (**: p<0.001). Note the significant increase in the association of miR-709 and MSE with H3K27me3 silencing complexes following *in vivo* nerve injury.

So far we have demonstrated that miRNAs employ a combinatorial mechanism for regulation of translation following PNS injury. This is particularly prominent for Egr2 protein the expression of which is repressed (Fig. 1A) through a complex pattern of regulation by opposing miRNAs (Fig. 2, 3 and 4). In most cases, destabilization of mRNA usually comprises the major component of repression while some targets are repressed without detectable changes in mRNA levels [36]. Since Egr2 is a transcription factor with a crucial role during myelination [37] we wanted to examine the possibility that the mRNA levels of Egr2 following sciatic nerve injury are regulated by miRNAs through transcriptional gene silencing. This would solidify the role of miRNAs as the central epigenetic regulators of the translational and transcriptional responses that characterize the cellular response to injury in the PNS. To perform this study, we first employed RT-PCR to examine the mRNA levels of Egr2 24 hours and 48 hours after sciatic nerve injury and compare this to the Egr2 mRNA expression in the uninjured nerve. This showed that expression of Egr2 mRNA is completely inhibited 48 hours after sciatic nerve injury (Fig. 5A), which agrees with the complete repression of Egr2 protein expression at the same time interval (Fig. 1A). However, RT-PCR fails to differentiate between decrease in transcription due to less efficient initiation (as in transcriptional gene silencing) versus degradation of mRNA (as in post-transcriptional silencing). As potent extinguishers of pre-existing programs [20], [23], miRNAs in injury must function to suppress myelination program prior to initiating dedifferentiation of Schwann cells. To specifically address the role of miRNAs in transcriptional silencing of Egr2 we performed a STarMir search for potential miRNA binding sites on the Myelin Specific Element (MSE) of the Egr2 promoter, which controls the expression of Egr2 in Schwann cells [38]. This showed that the MSE has efficient binding sites for miR-709, 468, 146b, 711 and 690 (Table 2). We selected miR-709 for our subsequent studies regarding the transcriptional gene silencing of Egr2 since STarMir predicted the highest total energy for miRNA 709 - MSE interaction (Table 2) indicating the possibility of a functional interplay. We next performed nuclear “run-on” assay by transfecting miR-709 into rat Schwann cells activated for Egr2 expression with ascorbic acid. Supporting our prediction, exogenously added miR-709 leads to transcriptional gene silencing of Egr2 as indicated by the decrease in the nascent transcripts of Egr2 (Fig. 5B), suggesting a *trans*-regulatory role for miR-709 to induce transcriptional silencing in injury in addition to the observed post-transcriptional gene silencing (Fig. 1A & 4C & D). Down-regulation of Egr2 protein expression in Schwann cells transfected with miR-709 (Fig. 5C) is consistent with the post-transcriptional and transcriptional silencing observed with luciferase assays and nuclear run-on assays respectively. Similar effect was observed when miR-138 was inhibited with antimiR-138 confirming our hypothesis that several miRNAs act synergistically to regulate the master regulator of myelination Egr2. It is possible that more miRNAs could be involved *in vivo* to shut down Egr-2 transcription more effectively during the process of injury response.

**Table 2:** STarMir analysis of accessibility of miRNA binding sites in targets (ΣΔG total, Kcal/mol).

Gene	miR-138	miR-709	miR-146b	miR-468	miR-690	miR-711
Egr2-MSE	−6.96	**−16.3**	−14.34	−13.93	−6.3	−14.8
		**−14.4**	−9.2	−6.34	−3.1	−10.5
		**−10.6**	−8.8	−4.5		−9.1
		**−9.44**	−8.2			−9.1
			−7			−5.86

A value less than -10 Kcal/mol means efficient interaction.

* miR-709 shows the most efficient binding sites in the MSE region of the Egr2 promoter.

Recent research suggests that miRNAs play a role in the regulation of genes at the chromatin level by affecting DNA methylation and histone post-translational modifications [4], [5], [8], [9]. To examine if DNA methylation contributes to repression of Egr2 transcription after sciatic nerve injury *in vivo*, we performed methylation PCR using primers specific for the CpG islands of the Egr2 proximal promoter. This revealed that the Egr2 proximal promoter is not subject to DNA methylation (Fig. 5D). Since miR-709 regulates nascent transcription of Egr2 we hypothesized that miR-709 contributes to the formation of epigenetic silencing complexes on Egr2 promoter with the repressive histone H3K27Me3 and Ago-1 to induce transcriptional silencing. Transcriptional gene silencing by miRNAs involving Ago proteins with or without the requirement of histone modifications has been shown previously [5]. To verify our hypothesis for the role of miR-709 in transcriptional silencing of Egr2 following peripheral nerve injury we performed *in vivo* ChIP with H3K27Me3 antibody using chromatin from mouse sciatic nerves. This revealed that 48 hours post-injury, Ago-1 forms complexes with H3K27Me3 (Figure 5E). These repressive complexes contain miR-709 (Figure 5F) and associate with the MSE of the Egr2 promoter *in vivo* (Figure 5F). RNase H treatment of lysates from injured and control nerves resulted in a decrease in the co-precipitation of miR-709 with the H3K27me3-MSE complex (Fig. 5F) while the association was unaffected by RNase A treatment (data not shown) indicating a direct interaction between miR-709 and the MSE promoter DNA. Collectively, our results demonstrate for the first time a functional role of miRNAs in transcriptional silencing of Egr2 promoter that affects its nascent transcription, as well as the association of Egr2 with epigenetically regulated silencing complexes *in vivo*. Since this transcriptional repression is epigenetically regulated it can be dynamically modified by extracellular cues and signaling cascades, which can explain the intrinsic potential of the PNS to regenerate.

## Discussion

Mammalian miRNAs directly repress hundreds of genes albeit each to a modest degree [36], but the efficacy of miRNA-mediated repression increases with the number of sites [39]. As expected ID-2 and p75^NTR^ mRNAs that show binding sites solely for miRNAs that are repressed in injury (RNA22 analysis) exhibit an increase in translation following the derepression of miRNAs (Fig. 1A). Intriguingly, translation of Sox-2, Nanog and c-Jun is upregulated, while Egr2 is repressed although these mRNAs carry binding sites for both up- and down-regulated miRNAs in injury (Fig. 2). This indicates that same clusters of miRNAs regulate transcripts differently depending on environmental cues. For instance, during development, miR-138 and miR-338 lead to the repression of Sox-2, c-Jun and other anti-myelinating factors to initiate differentiation of glial cells [19], [23] while these transcripts are translated following peripheral nerve injury despite having sites for other upregulated miRNAs (Fig. 2). Obviously, other context factors, such as local AU-content, RNA binding proteins, RNA tertiary structure, and the compact but multiple competing conformations of arbitrary RNA sequences [40] influence the accessibility of miRNA binding sites (Table S1).

In support of our data regarding the combinatorial role of various miRNAs during injury response, a recent paper described that a group of miRNAs modulates Schwann cell responses after nerve injury and that miR-140 affects Egr2 expression and myelination in an *in vitro* DRG/Schwann cell co-culture system [41]. Here, with our work we show that a distinct group of miRNAs regulates *in vivo* post-transcriptional gene silencing of the main factors that regulate injury response (Egr2, Sox-2, c-Jun, Nanog) through functional complexes with the Ago-2 protein in the cytoplasm of Schwann cells. Overexpression of miR-709 and/or an inhibitor of miR-138 (antimiR-138) lead to down-regulation of Egr2 protein expression in Schwann cells. This confirms our hypothesis that the cohort of miRNAs in peripheral nerve injury act in a concerted manner to modulate the cellular response. MiR-138 is upregulated during Schwann cell differentiation where suppression of anti-myelinating factors is a pre-requisite for the induction of promyelinating factors like Egr2. We also show that miR-709 orchestrates transcriptional gene silencing of Egr2 through direct interaction with the Egr2 promoter that guides the promoter to associate with H3K27me3-mediated silencing complexes *in vivo*. The actual outcome of this synchronous and concerted function of miRNAs results in a robust repression or derepression of translation as indicated by the protein levels of the miRNA-targeted transcripts (Fig. 1A & B). Undoubtedly, the regulation by miRNAs may not be the sole mechanism that controls gene expression however it ensures the extent of gene expression.

**Figure 6:**
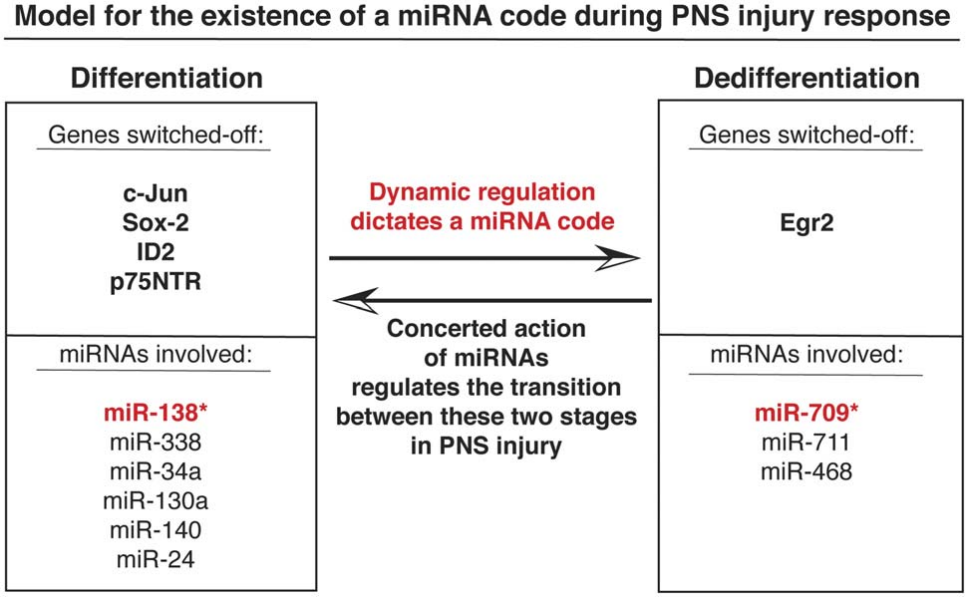
Schematic representation of our proposed model regarding the existence of a miRNA code, which regulates the transition between differentiation and dedifferentiation following *in vivo* PNS injury. The synchronous and concerted binding of miRNAs generates a unique code, which is dynamically regulated and affects the expression of the genes that modulate injury response in the PNS (e.g. c-Jun, Sox-2, Egr2).

Adding a new dimension to these complex interactions is the regulation of transcriptional gene silencing by miRNAs [4], [5], [8], [9]. Some epigenetic modifications, such as DNA methylation, provide more stable if not permanent repression of gene transcription that may even be inherited from one cell generation to the next [1]. Other modifications, such as histone methylation and acetylation are more labile and mediate reversible regulation of gene expression. During the course of PNS injury there is a gradual transition of Schwann cells from differentiation to dedifferentiation and *vice versa*, which means that regulation of gene expression must be dynamic and plastic. Our studies implicate miRNAs in achieving this plasticity by transiently recruiting the promoter of Egr2 into silencing complexes as indicated by the association of H3K27Me3 with Ago-1 at the MSE promoter. Moreover, our data support the conclusion that Argonaute proteins link the pathways for transcriptional and post-transcriptional gene silencing in the PNS as shown by the expression and association of both Ago-1 and Ago-2 with miRNAs in sciatic nerves. How miRNAs access Egr2 promoter sequences in the context of the living organism is currently under investigation. Since the miRNAs with binding sites within the MSE region of Egr2 are not encoded from the same genomic region it means that they do not target the opposite strand *in cis* as shown before [42]. Despite this, the fact that miR-709 represses the transcription of nascent Egr2 transcripts (Fig. 5B) means that there is a mechanism to recruit miRNAs to accessible promoter sequences. In addition, it is possible that miR-709 binds in *trans* and regulates the antisense strand of Egr2 promoter since MSE shows miRNA binding sites in both strands. It is beyond doubt however, that miR-709 interacts directly with the MSE-promoter DNA as indicated by the susceptibility of the complex to RNase H treatment (Fig. 5F). A schematic model depicting the concerted action of miRNAs during the dynamic transition from Schwann cell differentiation to dedifferentiation following PNS injury is presented in Fig. 6. With this model we want to introduce our hypothesis that binding of specific combinations of miRNAs to targets in precise concentrations and in synchronous fashion forms a dynamically regulated miRNA binding code that dictates whether a transcript is repressed or not during a cellular process or phenotypic outcome.

In conclusion, we describe for the first time that a discrete cohort of miRNAs act in concert to repress differentiation and myelination regulators such as Egr2 at different tiers of gene expression. They also coordinate the activation of dedifferentiation factors like Nanog, Id2, Sox-2 and C-Jun proving the conjecture that miRNAs have dual functions in gene expression tuning and expression buffering, and that both of these contribute to stabilizing homeostasis [43]. Our data reveal a set of specific miRNAs as the major endogenous triggers for transcriptional and post-transcriptional gene silencing that in turn orchestrate tissue remodeling and regeneration in the nervous system.

## Materials and Methods

### Ethics Statement

All animal work was performed according to institutional guidelines after approval of our animal protocol from the Institutional Animal Care and Use Committee (IACUC).

### MicroRNA Expression Profiling

Adult female CD1 mice (30–40 g) were anesthetized with isoflurane (2–3% in oxygen) according to the approved IACUC institutional protocols. Using aseptic technique, an incision was made on the upper thigh to expose the sciatic nerve. The nerve was cut ∼0.5 cm distal to the sciatic notch, and the wound closed with sterile surgical clips. For controls, the sciatic nerve was exposed and the wound closed without inducing any injury to the nerve. Total RNA was isolated from control (uninjured) and the distal stumps of axotomized sciatic nerves at 6 h and 24 h post injury using Trizol and Norgen RNA kit. Nerves from a total of 15 animals per experiment were used and the experiment was repeated twice. miRNA-microarrays were conducted by Exiqon (Vedbaek, Denmark). The samples were labeled using the miRCURY Hy3/Hy5 power labeling kit and hybridized on the miRCURY LNA Array (version.9.0). Log median ratios of the miRNAs expressed in 24h-injured nerve compared to the control were plotted.

### Prediction of miRNA Target Regions within Genes

RNA 22 (http://cbcsrv.watson.ibm.com/rna22.html) [29] was used to analyze the binding sites of individual microRNA within genes of interest (Egr2, Sox-2, QKI-6, c-Jun, ID-2, P75^NTR^ and Nanog) and their UTRs. Putative miRNA binding site(s) for each individual target gene were detected with a minimum 6 nt seed nucleus, zero unpaired nucleotides (nts) and -25 Kcal/mol maximum folding energy for the duplex of 14 nucleotides as constraints.

The effect of mRNA secondary structure on miRNA binding site availability was calculated using STarMir which models miRNA-target hybridization as a two-step process: nucleation involving 4 consecutive complementary nucleotides in the two RNAs and elongation of the hybrid to form a total a stable intermolecular duplex [28]. It calculates the total energy (ΣΔG_total_) of the interaction and value of -10 kcal mol^-1^ or less separates efficient interactions from inefficient interactions.

### MiRNA : Target Luciferase Reporter Assays

Selected miRNA (miR-138 and miR-709) binding regions of the genes of interest were amplified from mouse sciatic nerve RNA or from vectors (Sox-2 expression vector from Origene) cloned downstream of Luciferase gene in pmiR-report vector (Ambion) using the following primers. Egr2-Fwd-GCGACTAGTTT GACCAGATGAACGGAGTG; Egr2-Rev-GCGAAGCTTATCTCACGGTGTCCT GGTTC; Sox2-Fwd-GCGGAGCTCATGTATAACATGATGGAG, Sox-2-Rev-GCGTTTAAACCATGTGCGACAGGGG; C-Jun-Fwd: TGAGAACTTGACTGGTT GCG; C-Jun Rev: GAGGTTGGGGGCTACTTTTC. Cos-7 cells were transfected with individual vectors containing gene of interest with multiple predicted miRNA binding sites in the 3′-UTR of the luciferase gene in the pmiR-Report (50 ng) in presence or absence of 5 nM microRNA oligonucleotides, (anti-miR miRNA-138 or anti-miR miRNA-709, or a negative control oligo, Ambion or 25 ng of pCMV-vectors expressing miR-709 or miR-138). Transfections were performed in triplicate and repeated three times. Cell lysates were assayed for luciferase and β-Gal expression 24 h post transfection using Dual-Luciferase assay kit and β-galactosidase assay system (Promega). Transfection efficiency was normalized with β-gal report vector supplied with the pmiR-report kit (Ambion). Normalized Luciferase units were plotted and error is expressed as s.d.

### Northern Analysis and Quantitative RT-PCR of miRNA Expression

Total RNA extracted (5 µg) from the distal stumps of injured and uninjured nerves (n = 10) was fractionated on a denaturing 12% polyacrylamide gel containing 7 M urea, and transferred to Hybond N^+^ membrane (Amersham Pharmacia Biotech, USA) in 1× TBE buffer and cross-linked with UV (1200 µJ/cm^2^/sec) according to the manufacturer’s instructions. Denatured Biotin labeled miRCURY™ probes (5 pmols) were hybridized to the membrane in the UltraHyb (Ambion) buffer at 45°C overnight. The membrane was then washed, blocked and incubated with Streptavidin-Alkaline Phosphatase conjugate and developed with CDP Star reagent according to manufacturer’s instructions (Ambion Brightstar Biodetect kit) and was exposed to X-ray film for 2 h or overnight. Real time PCR analysis of microRNAs from control and 24 h post injury distal nerves was performed using Applied Biosystems miRNA RT-PCR kit. Individual microRNAs (24, 27b, 34a, 138, 146b 204, 338-3p, 468, 690, 709, 711 and U6) were reverse transcribed with microRNA Multiscribe kit and amplified with FAM-MGB-NFQ Taqman probes on ABI 7500 Fast instrument as per manufacturer’s instructions (Applied Biosystems, USA). The data was normalized with U6 snRNA C_T_ value as internal control and fold difference (2^-ΔΔC^_T_) between injured and uninjured control nerves for each microRNA was plotted as log 2 median ratios and error is expressed as s.d.

### Methylation PCR Analysis

Methylation status of Egr-2 and Sox-2 gene promoter CpG islands were analyzed using Methyl-Profiler DNA Methylation qPCR primer assays according to manufacturer’s instructions (SA biosciences, MD). Briefly high quality genomic DNA was isolated from control and injured sciatic nerves (24 h PI). DNA (0.125 µg/reaction) was digested with methylation sensitive and methylation dependent restriction enzymes. Following digestion, the remaining DNA is quantified by real time PCR in each individual enzyme reaction using primers that flank a promoter (gene) region of interest. The relative fractions of (hyper) methylated, intermediate methylated and unmethylated DNA were subsequently determined by comparing the amount in each digest with that of a mock (no enzymes added) digest.

### Ago-2 Immunoaffinity Purification and RT-PCR

Mouse sciatic nerves (10 per set) from control and injured (24h-post injury) were homogenized in 500 µl Lysis buffer (150 mM KCl, 25 mM Tris-HCl pH 7.4, 5 mM EDTA, 0.5% Nonidet P-40, 5 mM DTT, 1 mM PMSF, protease and phosphatase inhibitors (SIGMA), 100 U/ml RNase OUT (Invitrogen) and incubated on ice for 30 min. The lysates were then passed through a 27G needle several times to lyse completely and spun at 4°C for 30 minutes at 14,000 RPM. Protein A/G beads were pre-equilibrated in lysis buffer (10×v/v) twice. Cell lysates from control and injured nerves containing equal concentration of protein were pre-cleared with Protein A/G beads for 30 min at 4°C. Then the supernatants were incubated with Ago-2 antibody/ IgG for 4 hrs at 4°C. Pre-equilibrated Protein A/G beads were added to each reaction and incubated further at 4°C for 1h. The beads were washed three times with 10× volume of lysis buffer for 5 minutes. Five percent of the beads were frozen for SDS PAGE analysis. RNA was extracted directly from remaining beads with 25∶24:1 phenol:chloroform: isoamyl alcohol (Ambion). Trace amounts of phenol were removed by chloroform extraction and RNA was precipitated using sodium acetate with Glyco-Blue (Ambion) as a carrier. DNase treated RNA was precipitated and resuspended in RNase free water. cDNA was synthesized from a fraction of this RNA using oligo-dT primer and Superscipt II kit (Invitrogen) and genes such as Egr2, QKI-6, Sox-2, Nanog, ID-2 and c-Jun were amplified by PCR. For MicroRNA analysis, cDNA was prepared from the remaining RNA with individual microRNA RT-primer and then assayed by real-time PCR as per vendor’s instructions (Applied Biosystems). The data was normalized with miR-709 C_T_ value as internal control and fold difference (2^-ΔΔC^_T_) between injured and uninjured control nerves for each microRNA was plotted as log 2 median ratios and error is expressed as s.d.

### Immunoblotting

SDS Protein lysates isolated from control and Injured (48h-post injury) mouse sciatic nerves (n = 4) were separated on SDS-PAGE and probed with antibodies for Egr2 (Covance), Sox-2, c-Jun (cell signaling), ID-2 (Cell signaling), Nanog (R&D) and QKI-6 (Millipore), P75NTR (Abcam), Ago1, Ago2 and Dicer (Cell Signaling).

### Egr2 Regulation by miRNA in Rat Schwann Cells

Rat Schwann cells (1×10^6^) were nucleofected (Amaxa/Lonza) with 100 nM miR-709 or AntimiR-138 or both to confirm that these miRNA do indeed regulate Egr2 expression in Schwann cells. Lysates were made from transfected cells 48h post-transfection and were immunoblotted with Anti-Egr2 antibody. For controls untransfected and cells transfected with 100 nM control miRNA (non-targeting) were used.

### Nuclear Run-on Assay

Nuclear run-on assays were performed as described in [44]. Rat Schwann cells were nucleofected with control or miR-709 duplexes (Ambion) at 50 nm following pre-treatment with Ascorbic acid (50 µg/ml) for 48 h to activate Egr2 expression. Ascorbic acid was added 12 h post-transfection for a further 48 h and 1×10^7^ cells were washed with cold PBS, harvested and lysed on ice in 0.5% Nonidet-NP40 lysis buffer (10 mM Tris.HCl (pH 7.4), 10 mM NaCl, 3 mM MgCl_2_), and centrifuged at 500×g for 10 min. Supernatants were removed and nuclei were incubated in reaction buffer (10 mM Tris.HCl (pH 8.0), 5 mM MgCl_2_, 0.3 mM KCl) and 2.5 mM NTP plus Biotin-16-UTP mix (Roche) for 45 min at 30°C. The transcription reaction was stopped with Trizol and total nuclear RNA was isolated. Biotinylated nascent RNA transcripts were isolated by incubation with Streptavidin beads (Active Motif) for 2 h at room temperature on a rocking platform. Beads were collected by centrifugation and washed once with 2× SSC-15% formamide for 10 min and twice with 2× SSC for 5 min on a rocking platform. Biotinylated RNA was eluted from Streptavidin beads in Rnase free water by incubation at 90°C for 10 min and analyzed by qRT-PCR using Egr2 and GAPDH primers (Applied Biosystems). The data was normalized with GAPDH RNA C_T_ value as internal control and fold difference (2^-ΔΔC^_T_) between miR709 and control miR treated Egr2 expression in Schwann cells was plotted as log 2 median ratios and error is expressed as s.d.

### Sciatic Nerve *In vivo* CHIP

Sciatic nerves (5 per condition) from control and axotomized (distal nerves from 48 h post-injury) mice were isolated and snap frozen in methanol/dry ice bath and stored at -80°C. Nerves were then crosslinked with 1% Formaldehyde in PBS for 15 min at RT with rotation and neutralized with 0.3 M Glycine (final concentration) for 5 min at RT. Nerves were centrifuged at low speed and washed once with cold PBS plus protease and phosphatase inhibitors and homogenized in 600 µl SDS lysis buffer (ChIP Assay Kit, Millipore) supplemented with protease, phosphatase inhibitors (Sigma, MO) and RnaseOUT (Invitrogen) for 15 sec at Setting 3 (ProScientific Inc.). Cell lysates were sonicated on ice using Misonix Sonicator 3000, for 9–10 cycles of 30 sec ON and 1 min 30 sec Off at 80% power to shear DNA to lengths between 200 and 1000 base pairs being sure to keep samples ice cold. An aliquot of lysate (50 µl) was reverse crosslinked with the addition of 2 µl 5 M NaCl at 65°C for 4 h. The sample was then proteinase K treated for 1 h at 45°C and DNA was recovered with Phenol/Chloroform extraction and the efficiency of shearing was visualized on an agarose gel. Immunoprecipitations were carried out with precleared lysates using H3K27Me3 CHIP grade antibody (Millipore) or without any antibody for control following manufacturers protocol (Millipore). Lysates were either treated with RNase H (10 U) or RNase A (20 µg) prior to immunoprecipitations. Recovered antibody/antigen/DNA/miRNA complexes were washed as per the instructions and were used for western blot with H3K27Me3 (Millipore) and Ago1 (Cell signaling) antibodies as well as DNA/miRNA qPCRs. For DNA and miRNA analysis the immunoprecipitate was reverse crosslinked, proteinase K treated and extracted with phenol/chloroform. Recovered material from input (50 µl of lysate), no Ab and H3K27Me3 CHIP was used to perform realtime pCR of MSE promoter element using (MSE-F:CAACCACAGGCACCTCTCCGGG; MSE-REV: CAAGCCCCCACTGTTGGGCAGA) with SYBR-Green reagents (Quantas Biosciences) on Applied Biosystems instruments. For microRNA analysis, cDNA was made using miR-709 RT-primer (Applied Biosystems) following manufacturers instructions, cDNA reactions without reverse transcriptase were used as controls. miR-709 was amplified using Taqman real time PCR primer probe set (Applied Biosystems). Relative enrichment of DNA / miR-709 sequences was calculated by normalizing averaged cycle threshold (Ct) values to the input DNA, then calculating the difference between the normalized Ct values of injury and control nerves. These values were then transformed by 2^–n^, where n equals the net Ct value. Error is expressed as s.d.

## Supporting Information

Figure S1 **Taqman qRT-PCR analysis of individual microRNAs from control and 24 hour post-injury distal nerves, normalized with U6 snRNA as internal control.** Fold difference (2^-ΔΔCT^) between injured versus normal nerves is plotted using standard deviation as error.
(TIFF)

Figure S2 **Mature miRNAs 138, 690, 711 and U6 snRNA loading control were detected by Northern Blotting between control and in injury.**
(TIF)

Figure S3 **Cos-7 cells were transfected with empty pmiR-Report luciferase vector (50 ng) in presence of pCMV-vectors expressing miR-709 or miR-138 or empty pCMV vector and β-Gal report vector (25 ng each).** Transfections were performed in triplicate and repeated three times. Cell lysates were assayed for luciferase and β-Gal expression 24h post transfection using Dual-Luciferase assay kit and β-galactosidase assay system (Promega). Transfection efficiency was normalized with β-gal report vector supplied with the pmiR-report kit (Ambion). Normalized Luciferase units were plotted and error is expressed as s.d.
(TIF)

Table S1 **Accessibility analysis for miRNA binding sites in various gene targets using STarMir.**
(XLSX)

Table S2 **Accessibility analysis for miRNA binding sites in Luciferase constructs for Egr2, Sox2 and c-Jun using STarMir.**
(XLSX)
