# Supplementary figures and images for: Combinatorial Action of miRNAs Regulates Transcriptional and Post-Transcriptional Gene Silencing following in vivo PNS Injury
Source: PLoS One. 2012 Jul 6;7(7):e39674. doi: 10.1371/journal.pone.0039674 (PMC3391190; doi:10.1371/journal.pone.0039674)

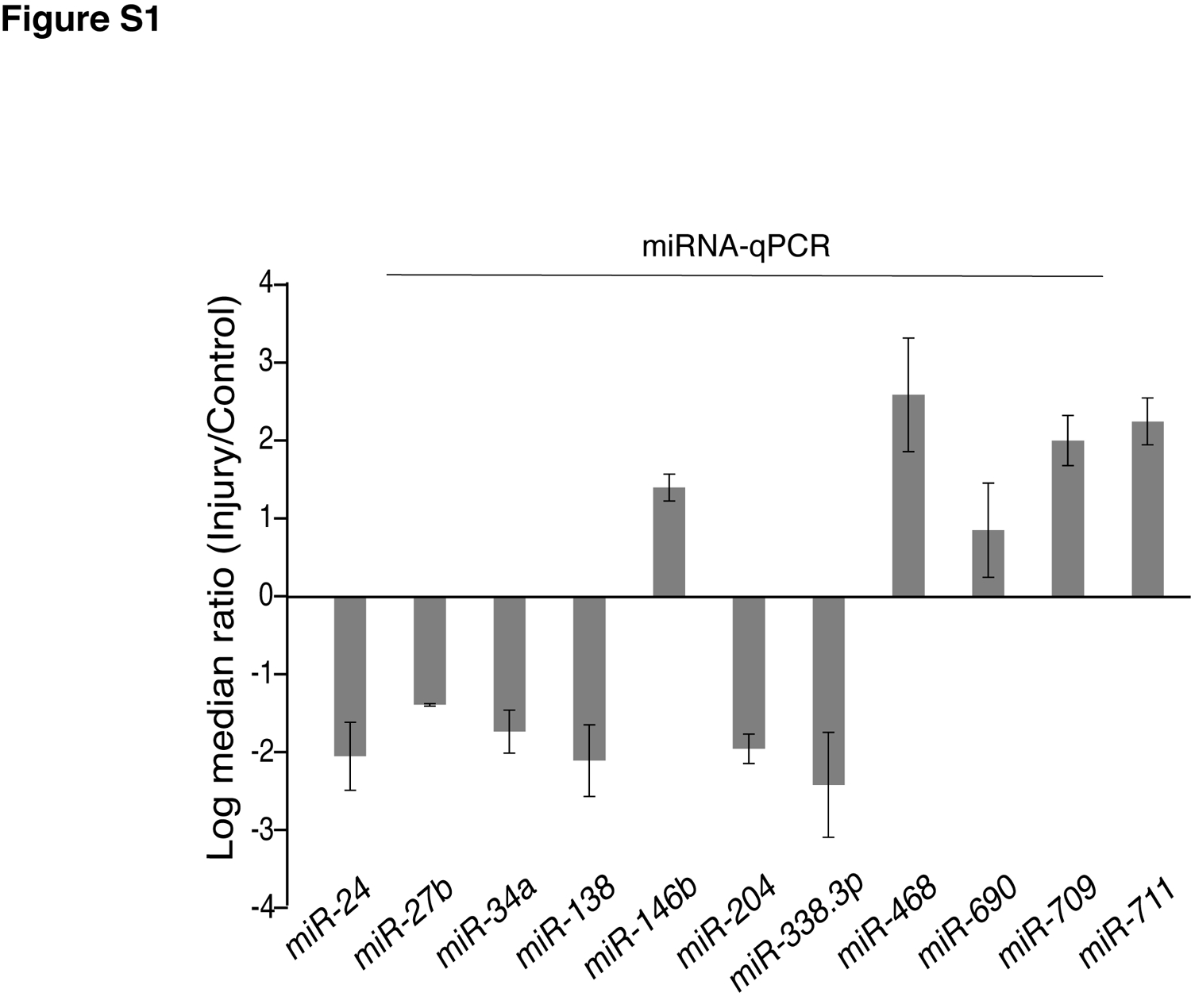

Supplement: Figure S1 — Taqman qRT-PCR analysis of individual microRNAs from control and 24 hour post-injury distal nerves, normalized with U6 snRNA as internal control. Fold difference (2-ΔΔCT) between injured versus normal nerves is plotted using standard deviation as error. (TIFF) [file pone.0039674.s001.tif]

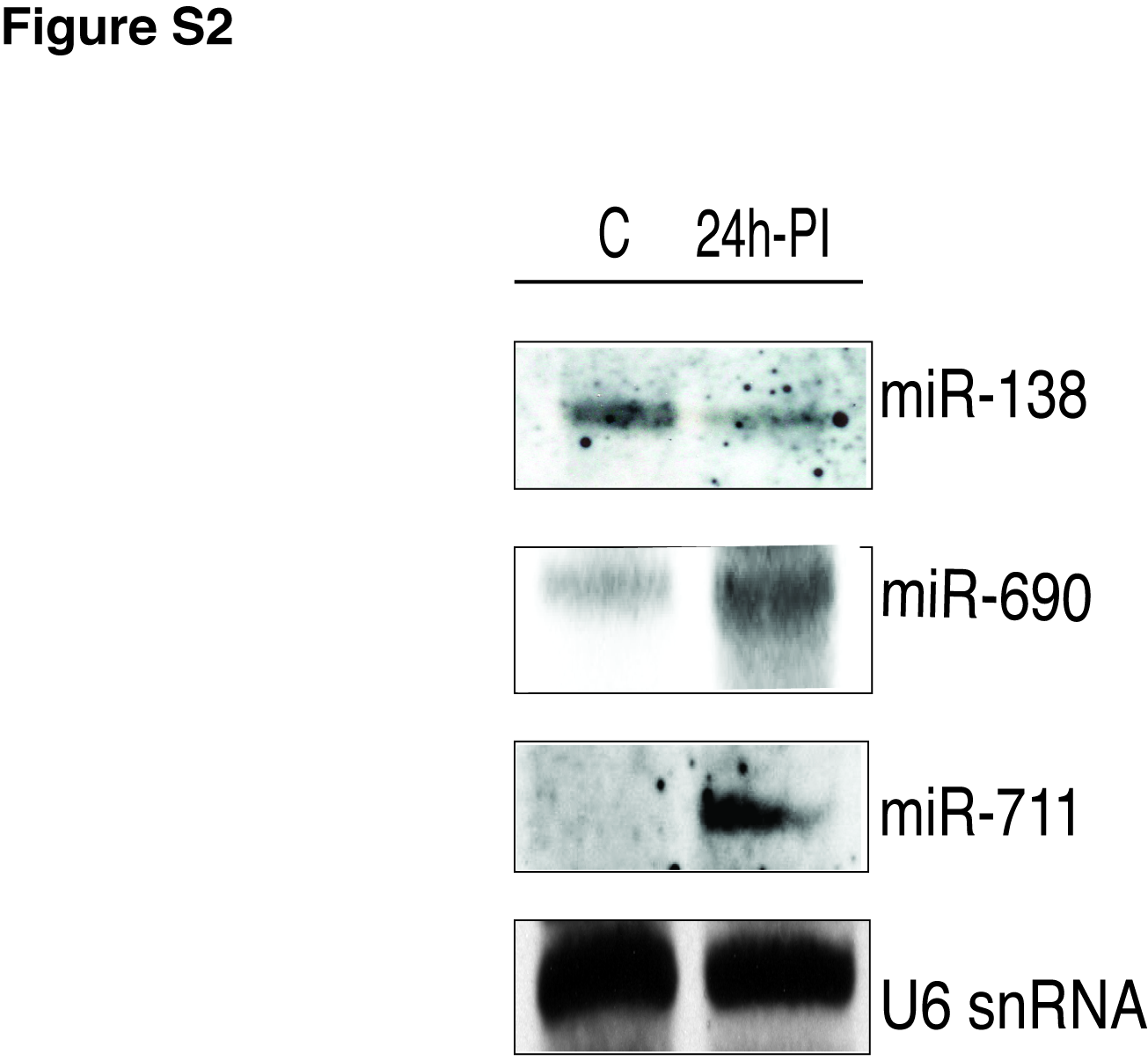

Supplement: Figure S2 — Mature miRNAs 138, 690, 711 and U6 snRNA loading control were detected by Northern Blotting between control and in injury. (TIF) [file pone.0039674.s002.tif]

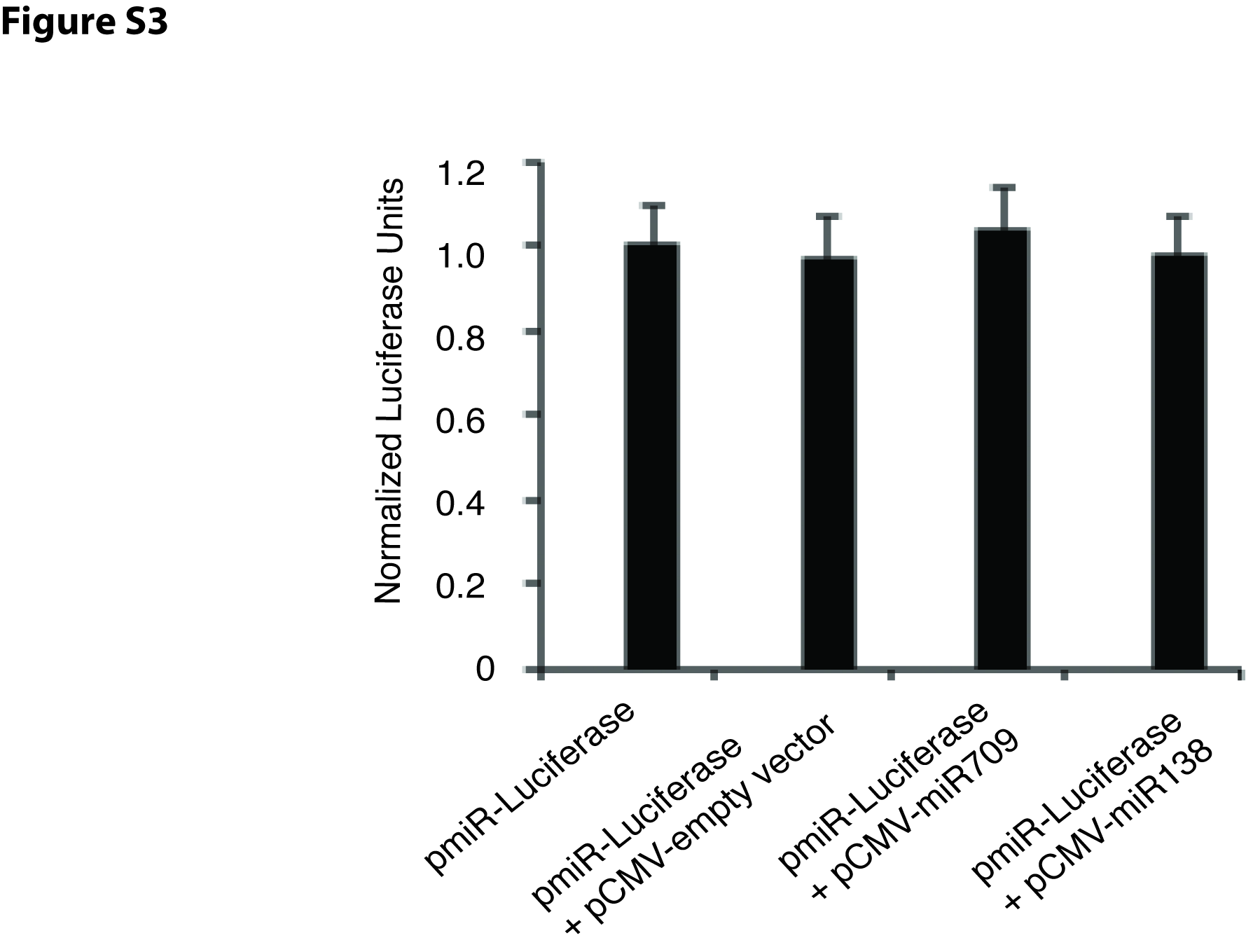

Supplement: Figure S3 — Cos-7 cells were transfected with empty pmiR-Report luciferase vector (50 ng) in presence of pCMV-vectors expressing miR-709 or miR-138 or empty pCMV vector and β-Gal report vector (25 ng each). Transfections were performed in triplicate and repeated three times. Cell lysates were assayed for luciferase and β-Gal expression 24h post transfection using Dual-Luciferase assay kit and β-galactosidase assay system (Promega). Transfection efficiency was normalized with β-gal report vector supplied with the pmiR-report kit (Ambion). Normalized Luciferase units were plotted and error is expressed as s.d. (TIF) [file pone.0039674.s003.tif]
